# Are NOACs as safe and efficient as VKA regarding thromboembolic prophylaxis and major bleeding in patients with surgical bioprosthesis and atrial fibrillation within 3 months of surgery?

**DOI:** 10.1093/icvts/ivab363

**Published:** 2022-01-03

**Authors:** Pedro Lamares Magro, Miguel Sousa-Uva

**Affiliations:** Department of Cardio-thoracic Surgery, Hospital de Santa Cruz, Carnaxide, Portugal

**Keywords:** Non-vitamin K antagonist anticoagulant, DOAC, Bioprosthesis, Anticoagulation

## Abstract

A best evidence topic in cardiac surgery was written according to a structured protocol. The question addressed was: ‘Are NOACs as safe and efficient as vitamin K antagonist regarding thromboembolic prophylaxis and major bleeding in patients with surgical bioprosthesis and atrial fibrillation within 3 months of surgery?’ Altogether more than *324* papers were found using the reported search, of which *6* represented the best evidence to answer the clinical question. The authors, journal, date and country of publication, patient group studied, study type, relevant outcomes and results of these papers are tabulated. The RIVER and ENAVLE trials showed non-inferiority of rivaroxaban (regarding mean time free from composite of death, major cardiovascular events or major bleeding at 12 months) and edoxaban (composite of death, clinical thromboembolic events or asymptomatic intracardiac thrombosis; and major bleeding) when compared with vitamin K antagonist. These studies include a low number of patients within 3 months of index surgery and overall low statistical power regarding this particular subgroup of patients. Data derived from lower evidence studies are compatible with the aforementioned findings. The available evidence suggests that non-vitamin K antagonist anticoagulants are as safe and as efficient as vitamin K antagonist regarding thromboembolic prophylaxis and bleeding event rates in patients with surgical bioprosthesis and atrial fibrillation within 3 months of bioprosthesis implantation. However, this evidence is derived from a limited number of studies with important methodological limitations. Expanding non-vitamin K antagonist anticoagulant recommendation to the early postoperative period warrants more confirmatory research.

## INTRODUCTION

A best evidence topic was constructed according to a structured protocol. This is fully described in the *ICVTS* [1].

## THREE-PART QUESTION

In [patients with surgical bioprosthesis and atrial fibrillation] is [non-vitamin K antagonist anticoagulant (NOAC) similar to vitamin K antagonist (VKA)] in terms of [safety (major bleeding) and efficacy (thromboembolic prophylaxis)] within 3 months of bioprosthesis implantation.

## CLINICAL SCENARIO

A 62-year-old aeroplane pilot with symptomatic aortic stenosis is proposed to surgical valve replacement. Because of his erratic schedules, the patient chooses a bioprosthesis in order to avoid therapeutic drug monitoring. However, in the postoperative period, the patient develops atrial fibrillation refractory to rhythm control attempts. You are tempted to use an NOAC to manage this patient postoperatively, however, your senior consultant reminds you that according to 2020 American College of Cardiology/American Heart Association (ACC/AHA) guidelines VKA should be considered for the first 3 months after bioprosthesis implantation. A review of the current evidence regarding this topic is warranted to validate either approach.

## SEARCH STRATEGY

A comprehensive literature review was performed searching Medline from inception to February 2021 using the PubMed interface. The following strategy was used: [(direct oral anticoagulants (DOAC) OR NOAC OR apixaban OR rivaroxaban OR edoxaban OR dabigatran) AND prosthesis]. References of selected papers were screened for additional relevant papers. The eligible papers were in English.

## SEARCH OUTCOME

In total, 324 papers were found using the reported search. From these, 6 papers were identified that provided the best evidence to answer the question. These are presented in Table 1.

**Table 1:** Best evidence papers

ARISTOTLE Guimarães et al [2] (2019) Clin Cardiol Multinacional Post hoc analysis of RCT (III)	156 patients with AF/Flutter AND ≥1 risk factor for stroke* AND history of bioprosthesis (n=104) or native valve repair (n=52) Apixaban=87 Warfarin=69 Timing of anticoagulation initiation: Unknown (not reported) All patients had AF/Flutter	Stroke or systemic embolism Major bleeding All-cause death	Apixaban:2.77% Warfarin:2.64% HR(95% CI):1.71(0.31-9.37) P=0.53 Apixaban:5.87% Warfarin:6.44% HR(95% CI):0.88(0.31-2.52) P=0.82 Apixaban: 4.61 Warfarin: 4.79 HR(95% CI): 1.02(0.34-3.04) P=0.98	
				
ENGAGE AF-TIMI 48 Carnicelli et al [3] (2017) Circulation USA Post hoc analysis of RCT (III)	191 patients with bioprosthesis (mitral=131; aortic=60) AND AF AND CHADS_2_ score≥2 Edoxaban (60mg):63 Edoxaban (30mg):58 Warfarin: 70 Timing of anticoagulation initiation: Unknown (date of surgery not collected) All patients had AF	Stroke or systemic embolic event Major Bleeding	Edoxaban 60mg vs Warfarin: HR (95% CI):0.37 (0.10-1.42); p=0.15 Edoxaban 30mg vs. Warfarin: HR (95% CI):0.53 (0.16-1.78); p=0.31 Edoxaban 60mg vs Warfarin: HR (95% CI):0.5 (0.15-1.67); p=0.26 Edoxaban 30mg vs Warfarin: HR (95% CI):0.12 (0.01-0.95); p=0.045	Does not include patients in the first 30 days after bioprosthesis implantation
				
RIVER Guimarães et al [4] (2020) NEJM Brazil RCT (II)	1005 patients with mitral bioprosthesis AND AF/Flutter Rivaroxaban: 500 Warfarin: 505 Timing of anticoagulation initiation: <3 months after surgery (18.8%) ≥ 3 months after surgery (79.8%) All patients had AF/Flutter	Mean time free from primary outcome (composite of death; major cardiovascular events, or major bleeding) At 12 months Death from cardiovascular causes or thromboembolic events Stroke Death Valve thrombosis Major bleeding	Rivaroxaban: 347.5 days Warfarin: 340.1 days RMST difference, 7.4 days; 95% CI: −1.4 to 16.3; P<0.001 for noninferiority P=0.10 for superiority Rivaroxaban:3.4% Warfarin:5.1% HR (95% CI):0.65 (0.35-1.20) Rivaroxaban:0.6% Warfarin:2.4% HR (95% CI):0.25 (0.07-0.88) Rivaroxaban:4% Warfarin:4% HR (95% CI):1.01 (0.54-1.87) Rivaroxaban:1% Warfarin:0.6% HR (95% CI):1.68 (0.40-7.01) Rivaroxaban:1.4% Warfarin:2.6% HR (95% CI):0.54 (0.21-1.35)	Patients in the warfa- rin group had an INR in the therapeutic range for a median of 65.5% of the time Only 18.8% of patients randomized in the first 3 months after surgery
				
ENAVLE Shim et al [6] (2021) J Thorac Cardiovasc Surg South Korea RCT (II)	218 patients with aortic (n=49%) or mitral (21%) bioprosthesis or mitral valve repair (n=39%) Edoxaban: 109 Warfarin: 109 Timing of anticoagulation initiation: Mean time 8 days after surgery AF: 61% of patients	Composite of death, clinical thromboembolic events or asymptomatic intracardiac thrombosis At 3 months Major bleeding	Edoxaban: 0%; Wafarin:3.7%; RD (95% CI): −0.0367 (–0.0720 to –0.0014; *P*<0.001 for noninferiority *Subgroup of AF patients:* Edoxaban: 0%; Warfarin:3%; RD: -0.0299; 95% CI: -0.0706 to 0.0109; *P*=0.003 Edoxaban: 2.8%; Warfarin: 0.9%; RD (95% CI): 0.0183 (–0.0172 to 0.0539); *P*=0.013 *Subgroup of AF patients*: Edoxaban: 3.1%; Warfarin: 1.5%; RD: 0.0158; 95% CI: -0.0352 to 0.0669; *P*=0.42	Small number of events
				
Pasciolla et al [6] (2020) Clin Drug Investig USA Retrospective cohort (III)	197 patients with bioprosthesis receiving anticoagulation after surgery NOAC:127 Apixaban:86 Rivaroxaban:40 Dabigatran:1 Warfarin:70 Timing of anticoagulation initiation: 4 days after surgery AF in 90% of patients	Major bleeding Thrombosis/stroke	NOAC:7.1% Warfarin:2.9% P=0.22 NOAC:2.4% Warfarin:0% P=0.20	Unmatched retrospective analysis Higher prevalence of CKD in the warfarin group
				
Nauffal et al [7] (2021) Ann Thorac Surg USA Retrospective cohort (III)	197 patients with bioprosthesis receiving anticoagulation after surgery NOAC:127 Apixaban:86 Rivaroxaban:40 Dabigatran:1 Warfarin:70 Timing of anticoagulation initiation: 4 days after surgery AF in 90% of patients	30-day mortality Rehospitalization for stroke/TIA Rehospitalization for major bleeding complications	OR: 1.08 95% CI: 0.80- 1.45 P=0.64 OR:0.94 95% CI:0.53-1.67 P=0.84 OR: 0.76 95% CI, 0.49-1.18 P=0.22	Multiple exclusion criteria including preoperative AF and preoperative anticoagulation Unmatched retrospective study
				
				

a Age ≥75 years, previous stroke/TIA, symptomatic heart failure, diabetes or hypertension.

CHADS_2_: congestive heart failure, hypertension, age ≥ 75 years, diabetes mellitus, stroke or TIA [2 points]; CI: confidence interval; HR: hazard ratio; NOAC: non-vitamin K antagonist anticoagulant; PSM: propensity score matched; RCT: randomized controlled trial; RD: risk difference; RMST: restricted mean survival time; RR: relative risk; TIA: transient ischaemic attack; VKA: vitamin K antagonist.

## RESULTS

The first hint about NOACs potential use in the postoperative period was given by *post* *hoc* analysis of the ARISTOTLE [2] and ENGAGE AF-TIMI 48 [3] trials. However, it is unclear to which extent both trials are truly representative of the population in the early postoperative period.

In the ARISTOTLE subanalysis, 156 bioprosthesis or valve replacement patients with atrial fibrillation (AF) and at least 1 risk factor for stroke were randomized to receive either apixaban or VKA with a follow-up of 1.6 years. Event rate including stroke [hazard ratio (HR), 1.71; 95% confidence interval (CI), 0.31–9.37; *P* = 0.53); major bleeding (HR, 0.88 95% CI, 0.31–2.52; *P* = 0.82) and all-cause death (HR: 1.02; 95% CI, 0.34–3.04; *P* = 0.98) were statistically similar between the groups. ENGAGE AF-TIMI 48 subanalysis compared 2 regimens of edoxaban with warfarin in 191 patients with bioprosthesis and moderate-to-high-risk AF, over a median follow-up of 2.8 years; revealing similar stroke or systemic embolic rates with both edoxaban regimes when compared to warfarin (edoxaban 60 mg versus warfarin: HR, 0.37; 95% CI, 0.10–1.42; *P* = 0.15; edoxaban 30 mg versus warfarin: HR, 0.53; 95% CI, 0.16–1.78; *P* = 0.31); but lower major bleeding rates with low-dose edoxaban (HR, 0.12; 95% CI, 0.01–0.95; *P* = 0.045) when compared with warfarin. Both analyses comprise a low number of patients and events and more importantly, both studies failed to report time of initiation of anticoagulation after surgery (with ENGAGE AF-TIMI 48 excluding patients in the first month after bioprosthesis implantation). Hence, it is probable that patients depicted in these trials are no representative of the focus of our best evidence topic, which is the early postoperative period.

More representative evidence is given by the RIVER and ENAVLE trials. In the RIVER trial [4], 1005 patients with mitral bioprosthesis and AF were randomized to receive either rivaroxaban (*n* = 500) or warfarin (*n* = 505). This multicentric open-label non-inferiority intention-to-treat design trial showed non-inferiority of rivaroxaban for the primary endpoint (mean time free from composite of death, major cardiovascular events or major bleeding at 12 months) (rivaroxaban, 347.5 days; warfarin, 340.1 days; difference, 7.4 days; 95% CI, −1.4–16.3; *P* < 0.001 for non-inferiority; *P* = 0.10 for superiority). Stroke rates at 12 months were statistically different, favouring rivaroxaban (rivaroxaban, 0.6%; warfarin, 2.4%; HR, 0.25; 95% CI, 0.07–0.88). Other secondary endpoints were similar between the true groups including major bleeding, valve thrombosis and death. However, because of the low number of some of these events, any findings should be interpreted with caution. Subgroup analysis showed that the difference between treatments regarding the primary endpoint was specially marked in patients randomized in the first 3 months after bioprosthesis implantation (rivaroxaban, 6.4%; warfarin, 18.9%; HR, 0.31; 95% CI, 0.12–0.79), favouring rivaroxaban. However, these results represent large CIs and lack *P* for interaction report, which renders their interpretation cumbersome.

Patients in the warfarin group had an INR in the therapeutic range for only a median of 65.5% of the time which can underestimate warfarin’s true effect. Study methodology permitted patient randomization at any time at least 48 h after surgery. Objectively, only 18.8% of the included patients were randomized in the first 3 months after surgery. This fact may result in misrepresentation of the early postoperative population in this cohort. However, it was also in the group of patients randomized in the first 3 months after surgery that the results favouring rivaroxaban were more evident, despite the aforementioned limitations.

The results of the ENAVLE trial were published by Shim *et al.* [5]. ENAVLE is a prospective open-label randomized controlled trial with a non-inferiority design which randomized patients to receive either edoxaban (*n* = 109) or warfarin (*n* = 109) during the first 3 months after bioprosthesis implantation (aortic = 49%; mitral = 21%) or mitral valve repair (39%). Sixty-one per cent of the included patients presented AF (60% vs 62%). The median time to initiation of anticoagulation was 8 days (interquartile range 7–10) after surgery. The intention-to-treat analysis demonstrated non-inferiority of edoxaban regarding the primary efficacy endpoint (composite of death, clinical thromboembolic events or asymptomatic intracardiac thrombosis) [edoxaban, 0%; warfarin , 3.7%; risk difference (RD), −0.0367; 95% CI, –0.0720 to –0.0014; *P *<* *0.001 for non-inferiority], as well as the primary safety outcome (major bleeding) (edoxaban, 2.8%; warfarin, 0.9%; RD, 0.0183; 95% CI, –0.0172 to 0.0539; *P *=* *0.013). The subgroup analysis of patients with AF is consistent with the main results regarding the primary efficacy endpoint (edoxaban, 0%; warfarin, 3%; RD, −0.0299; 95% CI, −0.0706 to 0.0109; *P *=* *0.003 for non-inferiority) and the primary safety outcome (edoxaban, 3.1%; warfarin, 1.5%; RD, 0.0158; 95% CI, −0.0352 to 0.0669; *P *=* *0.42); but no *P* for interaction of this subgroup is presented. This is a single-centre analysis including a small heterogeneous population and relatively large non-inferiority boundaries (8%) with limited statistical power, which precludes any definite conclusion without further research.

Further lower evidence observational studies corroborated the aforementioned findings: Pasciolla *et al.* [6] conducted a single-centre retrospective analysis of 197 patients who received a bioprosthesis and postoperative anticoagulation for 6 months after bioprosthesis implantation for any additional indication (>90% for AF). The median time to initiation of anticoagulation was 4 days (interquartile range 2–6) after surgery. This analysis included 70 patients receiving warfarin and 127 receiving an NOAC (apixaban: 86; rivaroxaban: 40; dabigatran: 1). During the follow-up period of 6 months after surgery, no statistical difference in major bleeding (NOAC: 7.1%; warfarin: 2.9%; *P* = 0.22) or thrombosis/stroke (NOAC: 2.4%; warfarin: 0; *P* = 0.20) rates were reported. Even though CHA2DS2 VASC and HAS-BLED scores were similar between the two groups this remains an unmatched retrospective analysis with inherent bias and small sample size.

Nauffal *et al.* [7] published the results of 26 522 patients from the Society of Thoracic Surgeons Adult Cardiac Surgery Database; who were anticoagulated at discharge (NOAC, 9769; warfarin, 16 753) for new-onset AF after a cardiac surgery procedure (including coronary artery bypass grafting and valvular procedures). In the overall cohort, there was no association between type of oral anticoagulant therapy and 30-day mortality (OR, 1.08; 95% CI 0.80–1.45; *P* = 0.64), rehospitalization rates for stroke or transitory ischaemic attack (OR, 0.94; 95% CI, 0.53–1.67; *P* = 0.84) or rehospitalization for major bleeding complications (OR, 0.76; 95% CI, 0.49–1.18; *P* = 0.22). The subgroup analysis of valvular patients revealed non-significant *P* for interaction for the 3 aforementioned outcomes (30-day mortality, *P* = 0.65; rehospitalization for stroke/transitory ischaemic attack, *P* = 0.12; rehospitalization for major bleeding, *P* = 0.19), thus, rendering the overall results applicable to the subgroup of valvular patients. This is an observational non-randomized study with inherent bias and multiple exclusion criteria including preoperative AF and preoperative anticoagulation. Additionally, the reported outcomes are limited to a 30-day follow-up period and events that required rehospitalization.

## CLINICAL BOTTOM LINE

The available evidence suggests that NOAC are as safe and as efficient as VKA regarding thromboembolic prophylaxis and bleeding event rates in patients with surgical bioprosthesis and AF within 3 months of index surgery. However, there is a significant lack of evidence regarding this specific period, as the available data may be influenced by the heterogeneity of pharmacological agents and bioprosthesis position/manufacturer used, timing of anticoagulation initiation, small and heterogeneous cohorts, and overall limited statistical power. Expanding NOAC recommendation to the early postoperative period calls for more confirmatory evidence, therefore, further research is warranted.

**Conflict of interest:** none declared. 

## Reviewer information

Interactive CardioVascular and Thoracic Surgery thanks Milan Milojevic and the other anonymous reviewers for their contribution to the peer review process of this article.
